# Disparities in mental health service utilization among adolescents with depression: Results from a 2022 U.S. National Survey

**DOI:** 10.1371/journal.pmen.0000388

**Published:** 2025-08-20

**Authors:** Su Chen Tan, Kaylynn Hunt, Brittany Shelton

**Affiliations:** 1 NSF Center for Analysis and Prediction of Pandemic Expansion, The University of Tennessee, Knoxville, Tennessee, United States of America; 2 Department of Public Health, The University of Tennessee, Knoxville, Tennessee, United States of America; PLOS: Public Library of Science, UNITED KINGDOM OF GREAT BRITAIN AND NORTHERN IRELAND

## Abstract

The prevalence of adolescent depression has increased following the COVID-19 pandemic, and adolescent depression is often under-treated. The emergence of new barriers resulting from the COVID-19 pandemic limited existing efforts to address pre-existing inequalities in appropriate mental health treatment utilization. We analyzed data from adolescents with major depressive episode (MDE) in the 2022 U.S. National Survey on Drug Use and Health to examine mental health service utilization by rurality, race/ethnicity, gender, age, health insurance coverage, and poverty level. We applied analytic weights to estimate nationally representative estimates and account for survey nonresponse. Multivariate logistic regression identified significant disparities in the use of mental health services. The 2022 NSDUH assessed adolescent MDE based on past-year self-reported depressive symptoms based on the Diagnostic and Statistical Manual of Mental Disorders (DSM-IV) criteria. Among the 19.2% of adolescents aged 12–17 who experienced MDE, only 47.5% received treatment within the past year. Adolescents in rural areas had significantly lower odds of receiving specialist treatment compared to their urban counterparts [adjusted odds ratio (AOR) 0.64 (95% confidence interval (CI) 0.47-0.87)]. Odds of receiving telehealth services were significantly lower for rural adolescents [AOR 0.64 (95% CI 0.44-0.93)] but were significantly higher for adolescents with insurance (public insurance [AOR 2.99 (95% CI 1.10-8.14)] and private insurance [AOR 3.82 (95% CI 1.39-10.49)]). Older adolescents had lower odds of utilizing school-based services than younger adolescents [AOR 0.52 (95% CI 0.38-0.71)]. Females had greater odds and Black adolescents significantly lower odds of utilizing any mental health treatment relative to males and non-Hispanic White adolescents, respectively [Females: AOR 1.59 (95% CI 1.11-2.28); Black: AOR 0.36 (95% CI 0.22-0.59)]. Our findings continue to illustrate the persisting inequity in mental health treatment among adolescents from marginalized groups amidst the COVID-19 pandemic. Tailored strategies to address these inequities are needed.

## Introduction

Adolescent depression is a public health concern with a prevalence has been steadily rising among adolescents in the United States since the COVID-19 pandemic [1]. Based on the data gathered from the National Survey of Drug Use and Health (NSDUH) [2], the rate of adolescent depression surged from 8.1% in 2009 to 19.5% in 2022 [1,3]. Although concerns about youth mental health existed prior to the COVID-19 pandemic, the past several years have subjected adolescents to various unprecedented challenges, including prolonged quarantines, school closures, interrupted social interactions, COVID-19 infections, the loss of loved ones, and an overarching sense of uncertainty in their daily lives [4]. Adolescent-onset depression is linked to more severe social and psychological consequences compared to depression that begins in adulthood. Early recognition and interventions are necessary to prevent long-lasting negative educational, health, and social outcomes [5–9]. There has been extensive research focused on the identification and treatment of mental health conditions in adolescents; however, the increased burden of mental disorders and the disruption of mental health services due to the COVID-19 pandemic created new challenges to the equitable delivery of quality services to those in need [10].

Adolescent mental health service utilization is shaped by a range of intersecting factors, including race, gender, geography, and health insurance coverage [11–13]. Historically, adolescents of African American/Black, Asian, and Hispanic descent encounter diminished access to mental health treatment, including prescription medication, consultations with mental health specialists or medical professionals, and outpatient therapy, compared to non-Hispanic White adolescents [11]. In addition, racial and ethnic minority groups faced a higher exposure to morbidity and mortality related to COVID-19 [14–17], increasing the mental tolls on communities of color and potentially contributing to the onset of or exacerbating psychological disorders, including depression [18–21]. Racial/ethnic minority groups are not the only sub-populations to experience a disproportionately high rate of depression. Among adolescents, depression is more prevalent in girls, who are also more likely than boys to receive a diagnosis and undergo treatment [12]. In rural areas, the distinct socio-cultural obstacles that can increase rural adolescents vulnerability to depression and anxiety are compounded by the shortage of mental health providers [13]. Although primary care providers typically manage mild to moderate anxiety and depression, rural areas often lack specialized clinicians to support them in connecting patients to therapy and treatment for more severe or complex mental health disorders [22]. Moreover, rural adolescents encounter greater challenges than their urban peers in their ability to access the technology and reliable internet connectivity essential for telehealth services, despite telehealth becoming increasingly crucial for ensuring effective and equitable mental health care delivery to individuals affected by distance and isolation, particularly during the COVID-19 pandemic [23]. The incidence of suicide among rural adolescents is nearly double that of urban areas, with untreated depression and anxiety identified as major risk factors [13]. Additionally, evidence suggests that uninsured adolescents, particularly those from racial minorities, have lower rates of mental health service utilization [24], highlighting the role of insurance coverage in reducing barriers to accessing necessary mental health care and addressing health disparities. In sum, the convergence of high depression rates with diminished access to care constitutes a medical and public health crisis.

Since the World Health Organization (WHO) and the U.S. Secretary of Health and Human Services declared the end of the global health emergency in May 2023, the number of studies on mental health service utilization and disparities during the COVID-19 pandemic has increased but remains limited, especially in relation to adolescent depression [25]. In an earlier study, Flores and colleagues analyzed the nationally representative 2021 NSDUH data and documented lower adolescent depression treatment rates among racial and ethnic minority groups [25]. Previous studies have highlighted disparities and national patterns in adolescents use of mental health services, influenced by gender, race/ethnicity, geographical area, and health insurance coverage [11,26–29]; however, to our knowledge, existing studies relied on data collected from before 2022 and may not fully capture the potential shifts in utilization patterns influenced by the COVID-19 pandemic. Therefore, updated analyses using more recent data are necessary to better understand current disparities and inform targeted interventions and policy changes. To thoroughly examine adolescent mental health service utilization patterns during the COVID-19 pandemic, we analyzed the National Survey of Drug Use and Health data [2] from 2022 to quantify and contrast mental health service utilization for adolescent depression in relation to race/ethnicity, age, urban/rural residence, and health insurance coverage. Our goal is to generate evidence highlighting inequitable access to mental health services among adolescents with major depressive episode (MDE), as well as the persistent structural disparities shaped by socioeconomic, geographic, and systemic factors. Understanding the inequity in mental health service utilization among adolescents is critical for identifying key areas that require targeted interventions and policies aimed at improving access to mental health services for disadvantaged populations, ultimately leading to better outcomes for those who are most in need.

## Methods

### Data

The 2022 NSDUH is a nationally representative cross-sectional survey that the Substance Abuse and Mental Health Services Administration (SAMHSA) administered in 2022 in all 50 states and the District of Columbia [2]. NSDUH is representative of the civilian non-institutionalized population aged 12 and older in the United States. The survey encompasses residents of various housing types, including houses, townhouses, apartments, and condominiums, as well as non-institutional group quarters like shelters, boarding houses, dormitories, halfway houses, migrant camps, and military bases. Homeless people who do not use shelters, military personnel on active duty, and residents of institutional group quarters, such as jails and hospitals, were excluded by NSDUH from participation in the survey. A multistage, stratified sampling design was used to select a nationally representative sample through sequential selection of geographical regions, census tracts, block groups, dwelling units, and individuals based on household age composition [2]. The 2022 NSDUH used multi-mode data collection, whereby the surveys were completed either in person or via the web [2]. Missing data in the 2022 NSDUH were addressed using the five multiply imputed datasets, which account for item-level nonresponse and enable unbiased parameter estimation by accounting for variability within and between imputations [2].

### Study population

All respondents aged 12–17 years old and classified by NSDUH as having a major depressive episode (MDE) in the past year were included in the study (n = 2283). NSDUH characterized adolescent respondents as having MDE in the past year if they had met the Diagnostic and Statistical Manual of Mental Disorders, Fifth Edition (DSM-V) criteria [2]. MDE is classified by a persistent depressed mood or a loss of interest and pleasure, occurring most of the day, nearly every day, for a minimum of two weeks. This is accompanied by at least four other symptoms, which may involve changes in appetite or weight, sleep issues, psychomotor agitation or slowing, fatigue, feelings of worthlessness or excessive guilt, reduced concentration, and recurring thoughts of death or suicide [2]. To assess these symptoms of MDE, adolescents were asked a series of questions from the depression section of the National Comorbidity Survey-Adolescent version [30]. The National Comorbidity Survey-Adolescent has been shown to provide nationally representative estimates of the prevalence and correlates of adolescent mental disorders, including clinical depression, among diverse populations [30].

### Outcome measures

The outcomes of interest were mental health service utilization regarding MDE treatment in the past year. Past year’s mental health service utilization was constructed as a dichotomous variable, whereby respondents received a score of 1 if they had received treatment and 0 if they had not. Those who reported receiving mental health treatment were asked to identify their sources of care. We defined specialist clinician treatment as care provided by a psychiatrist, psychologist, social worker, counselor, or other mental health professional (1 = received; 0 = not received). In addition, we assessed whether any psychotropic medication was prescribed for MDE (1 = received; 0 = not received). School-based mental health treatment included any care received from a school social worker, school psychologist, school counselor, or through specialized programs for students with emotional or behavioral issues. Additionally, we assessed telehealth-based mental health treatment utilization in the past year by examining respondents' treatment sources, counseling, or support accessed via the Internet or phone hotlines.

### Variables of interest

Variables of interest include age, gender, race/ethnicity, geographical area, health insurance status, and household income relative to the poverty level. The geographical area variable categorized respondents residences as large metropolitan, small metropolitan, or non-metropolitan/rural, using the 2013 U.S. Rural-Urban Continuum Codes [31]. Race and ethnicity were classified as non-Hispanic White (hereafter White), non-Hispanic African American/Black (hereafter Black), non-Hispanic Asian (hereafter Asian), Hispanic, and Others. Due to the small proportion (approximately 9%) of respondents identifying as non-Hispanic American Indian or Alaska Native, non-Hispanic Native Hawaiian or Pacific Islander, or non-Hispanic of multiple races, these groups were combined into the “Others” category.

The age variable was categorized into 12–13 years, 14–15 years, and 16–17 years. Similarly, gender was classified as either male or female. The household income variable is a categorical variable defined as household income below the federal poverty level (hereafter <100%), household income up to twice the federal poverty level (hereafter 100%-200%), and household income above twice the federal poverty level (hereafter >200%). The federal poverty level was determined by the 2022 poverty guidelines published by the U.S. Department of Health and Human Services. Health insurance status was constructed as a categorical variable that included private insurance, public insurance (including Medicaid, Children’s Health Insurance Program, Medicare), other health insurance (hereafter Other), more than 1 type of insurance (hereafter >1 type), and none (S1 Text).

### Data analysis

We first conducted descriptive analyses to outline the characteristics of respondents with MDE and mental health service utilization in the past year. We then examined univariate associations between types of mental health service utilization and relevant variables of interest using the Chi-squared test. Finally, adjusted multivariate logistic regression models were employed to assess the odds related to mental health service utilization, including any treatment, specialist clinician treatment, school-based services, prescription medication, telehealth services, and sample characteristics. Logistic regression analyses were performed and adjusted for age, gender, race/ethnicity, geographical area, health insurance status, and poverty level. Analytic weights were applied to model estimates to be nationally representative and account for survey nonresponse. All statistical analyses were conducted using SPSS version 29. Statistical significance was defined using a two-sided alpha of 0.05.

## Results

### Characteristics of U.S. adolescent with Major Depressive Episode (MDE) in the past year

In the 2022 NSDUH sample, 2283 out of 11969 respondents aged 12–17 (19.2%) had MDE in the past year. Using survey weights, the analysis revealed that the majority of the adolescents with MDE were female (71.0%). Self-reported race and ethnicity included 54.2% White, 11.9% Black, 24.9% Hispanic, 5.1% Asian, and 3.8% more than one race or ethnicity, Native American, Hawaiian or Pacific Islander (Table 1). Approximately one-fifth were 12–13 years old (22.6%), 39.6% were 14–15, and 37.8% were 16–17. Over half (58.1%) of the sample lived in large metropolitan areas, and more than half (59.7%) had a total household income of more than twice the federal poverty level. Only 2.7% had no insurance, 50.2% were privately insured, and 40.1% were publicly insured.

**Table 1:** Descriptive characteristics of adolescents with major depressive episode (MDE) in the past year.

Unweighted N = 2,283 Weighted N = 4,781,706	Weighted number	Weighted % (%)
Gender Female Male	3,396,840 1,384,866	71.0 29.0
Race Asian Black Hispanic Others White	244,248 569,212 1,191,367 183,585 2,593,294	5.1 11.9 24.9 3.8 54.2
Age 12-13 14-15 16-17	1,080,837 1,894,815 1,806,054	22.6 39.6 37.8
Area type Large metropolitan Small metropolitan Rural/non-metropolitan	2,278,841 1,469,632 533,232	58.1 30.7 11.2
Insurance type None Public Private Other > 1 type	129,851 1,915,471 2,402,761 81,597 252,026	2.7 40.1 50.2 1.7 5.3
Poverty level < 100% 100% - 200% > 200%	839,646 1,085,528 2,856,531	17.6 22.7 59.7

### Mental health service types by respondent characteristics

After applying analytic weights, 47.5% of the adolescents with MDE in the past year had received any treatment, 39.1% received specialist clinician treatment, 30.5% received school-based services, and 25.0% received prescription services (Table 2). Moreover, 34.5% of adolescents utilized telehealth services for mental health with MDE. The proportion of adolescents receiving any form of mental health treatment did not differ significantly across area types. Among all racial and ethnic categories, non-Hispanic White adolescents had the highest rates of treatment: 62.8% received any treatment, 62.1% received specialist clinician care, 64.5% utilized telehealth services, and 67.3% were prescribed medication (P < 0.001). The differences in receiving school-based services across racial groups were not statistically significant. In terms of gender, there was no statistically significant difference between male and female adolescents in utilizing any kind of mental health treatment. As for age, adolescents with MDE in the 14–15 age category utilized the highest percentage of telehealth services (39.6%; P < 0.01) compared to the 16–17 (30.8%) and 12–13 (29.6%) age categories. The proportion of adolescents with MDE utilizing other mental health services was not statistically significant across ages. The adolescents with MDE in the household income > 200% federal poverty level category utilized the highest percentage of telehealth services (67.8%; P < 0.01) compared to the 100% to 200% (18.4%) and <100% (13.8%) categories. As for health insurance status, adolescents with private insurance had the highest percentage of utilization of telehealth services (56%), followed by public insurance (36.4%), and those with more than one type of insurance (5.9%; P < 0.01). Similarly, private insurance holders had the highest proportion of using prescription medication (55.4% vs. public 37.7%: vs. none 1.0%: vs. more than 1 type 5.6%%; P < 0.05).

**Table 2:** Types of mental health service utilized during the past year by adolescents with major depressive episode (MDE)’s characteristics.

Unweighted N	Any treatment		Specialist clinician		School-based services		Telehealth			Prescription medication	
1097		872		668		789			594	
Weighted N	2,256,060 (47.5%)		1,829,842 (39.1%)		1,458,842 (30.5%)		1,649,575 (34.5%)			1182,671 (25.0%)	
Percentage	%	P-value	%	P-value	%	P-value	%	P-value	%		P-value
Gender Female Male	**75.4** **24.6**	0.020*	75.6 24.4	0.057	75.3 24.7	0.070	74.3 25.7	0.160	76.2 23.8		0.062
Race Asian Black Hispanic Others White	**3.2** **7.9** **22.8** **3.4** **62.8**	<0.001**	**3.9** **7.8** **22.9** **3.3** **62.1**	0.010*	5.8 5.6 26.5 3.4 58.7	0.070	**3.3** **7.4** **21.9** **2.8** **64.6**	<0.001**	**2.8** **6.3** **20.3** **3.3** **67.3**		<0.001**
Age 12-13 14-15 16-17	20.9 38.8 40.3	0.543	20.1 39.6 40.4	0.606	**29.6** **39.6** **30.8**	0.006**	20.0 43.0 37.0	0.309	20.9 38.3 40.9		0.566
Area type Large metropolitan Small metropolitan Rural/non-metropolitan	57.5 32.5 10.1	0.286	59.3 31.2 9.5	0.121	61.5 29.1 9.5	0.346	59.9 30.6 9.5	0.307	52.6 34.9 12.5		0.810
Insurance type None Public Private Other > 1 type	1.7 37.9 52.7 1.2 6.4	0.074	1.8 38.5 52.8 0.7 6.3	0.092	2.5 38.7 53.3 0.9 4.6	0.434	**1.0** **36.4** **56.1** **0.5** **5.9**	0.003**	**1.0** **37.7** **55.4** **0.3** **5.6**		0.024*
Poverty level < 100% 100% - 200% > 200%	15.8 21.9 62.3	0.360	15.3 12.3 62.4	0.393	15.2 22.1 62.7	0.409	**13.8** **18.4** **67.8**	0.010*	14.2 25.0 60.8		0.205

^a^*Significant at P < .05; **Significant at P < .01.

### Logistic regression analysis of mental health service utilization

Logistic regression showed that adolescents in non-metropolitan areas had lower odds of specialist clinician treatment (AOR 0.65; 95% CI 0.44-0.96) and telehealth services (AOR 0.65; 95% CI 0.43-0.98) compared to adolescents in large metropolitan areas (Table 3). No differences in school-based services and prescription medication utilization were observed across area types. Black adolescents were less likely to utilize school-based services (AOR 0.31; 95% CI 0.17-0.55) and less likely to receive specialist clinician treatment (AOR 0.41; 95% CI 0.24-0.69) than White adolescents. No statistically significant differences were observed among other racial/ethnic groups in the regression model. Black and Hispanic adolescents had lower odds of receiving prescription medication than non-Hispanic White adolescents, with AOR of 0.26 (95% CI 0.14-0.48) and 0.53 (95% CI 0.35-0.80), respectively. As a whole, Asian (AOR 0.30; 95% CI 0.13-0.70), Black (AOR 0.37; 95% CI 0.22-0.62), and Hispanic (AOR 0.62; 95% CI 0.41-0.96) adolescents had lower odds of receiving any mental health treatment than non-Hispanic White adolescents, but not Others adolescents (AOR 0.54; 95% CI 0.28-1.06).

**Table 3:** Logistic regression of mental health service utilization during the past year among adolescents with major depressive episode (MDE).

	Any treatment AOR (95% CI)	Specialist clinician AOR (95% CI)	School-based services AOR (95% CI)	Telehealth AOR (95% CI)	Prescription medication AOR (95% CI)
Gender Female Male	1.57* (1.11-2.21) Ref	1.52* (1.02-2.26) Ref	1.30 (0.96-1.77) Ref	1.32 (0.94-1.99) Ref	1.50* (1.02-2.21) Ref
Race Asian Black Hispanic Others White	0.30* (0.13-0.70) 0.37* (0.22-0.62) 0.62* (0.41-0.96) 0.54 (0.28-1.06) Ref	0.45 (0.20-1.03) 0.41* (0.24-0.69) 0.70 (0.46-1.08) 0.59 (0.31-1.13) Ref	0.96 (0.29-3.22) 0.31* (0.17-0.55) 0.94 (0.61-1.44) 0.73 (0.38-1.37) Ref	0.35* (0.15-0.83) 0.40* (0.22-0.70) 0.65* (0.45-0.94) 0.46* (0.24-0.92) Ref	0.41 (0.16-1.06) 0.26* (0.14-0.48) 0.53* (0.35-0.80) 0.72 (0.37-1.39) Ref
Age 12-13 14-15 16-17	Ref 1.11 (0.73-1.67) 1.34 (0.83-2.15)	Ref 1.14 (0.79-1.66) 1.31 (0.84-2.05)	Ref 0.67 (0.43-1.14) 0.52* (0.38-0.71)	Ref 1.40 (0.87-2.26) 1.22 (0.82-1.81)	Ref 1.10 (0.70-1.73) 1.34 (0.87-2.07)
Area type Large metropolitan Small metropolitan Rural/non-metropolitan	Ref 1.00 (0.75-1.33) 0.65* (0.44-0.96)	Ref 0.87 (0.66-1.15) 0.60* (0.42-0.84)	Ref 0.81 (0.58-1.14) 0.66 (0.38-1.14)	Ref 0.87 (0.68-1.11) 0.65* (0.43-0.98)	Ref 1.09 (0.77-1.55) 0.83 (0.56-1.25)
Insurance type None Public Private Other > 1 type	Ref 1.98 (0.86-4.56) 2.09 (0.87-5.05) 1.17* (2.61-5.22) 2.91 (0.86-9.84)	Ref 1.72 (0.71-4.44) 1.76 (0.70-4.42) 0.50 (0.09-2.70) 2.28 (0.66-7.86)	Ref 1.03 (0.37-2.86) 1.02 (0.36-2.88) 0.52 (0.13-2.05) 0.84 (0.27-2.61)	Ref 3.36* (1.26-8.93) 3.31* (1.22-8.98) 0.79 (0.11-5.59) 3.68* (1.08-12.57)	Ref 2.98 (0.83-10.76) 3.40* (1.01-11.44) 0.44 (0.07-2.92) 2.86 (0.70-11.70)
Poverty level < 100% 100% – 200% > 200%	Ref 0.92 (0.56-1.52) 0.92 (0.62-1.33)	Ref 1.02 (0.60-1.72) 1.01 (0.69-1.49)	Ref 1.16 (0.67-2.01) 1.16 (0.76-1.78)	Ref 0.90 (0.50-1.62) 1.31 (0.81-2.12)	Ref 1.25 (0.79-1.97) 1.00 (0.61-1.64)

^a^AOR, adjusted odds ratio; CI, confidence interval.

^b^Model adjusted for age, race and ethnicity, gender, geographical area, health insurance, and poverty level.

^c^*Significant at P < .05.

Compared to male adolescents, female adolescents were more likely to receive any mental health (AOR 1.57; 95% CI 1.11-2.21), specialist clinician treatment (AOR 1.52; 95% CI 1.02-2.26), and prescription medication (AOR 1.50; 95% CI 1.02-2.21), but not school-based services and telehealth services, compared to male adolescents. Relative to adolescents aged 12–13, adolescents in the 16–17 age group were less likely to utilize school-based services (AOR 0.52; 95% CI 0.38-0.71). The regression model revealed no statistically significant differences in the utilization of other mental health services across age groups. As for healthcare insurance status, compared to adolescents without insurance, adolescents with public insurance (AOR 3.36; 95% CI 1.26-8.93), private insurance (AOR 3.31; 95% CI 1.22-8.98), and more than 1 type of insurance (AOR 3.68; 95% CI 1.08-12.57) had higher odds of utilizing telehealth services; adolescence with private insurance had greater odds of receiving prescription medication (AOR 3.40; 95% CI 1.01-11.44); adolescents with “Others” insurance had higher odds of utilizing any treatment (AOR 1.17; 95% CI 2.62-5.22); no significant difference was found across the utilization of specialist clinician treatment and school-based services. There was no statistical difference between the odds of utilization of mental health services and household income with reference to the federal poverty level.

## Discussion

In analyzing the nationally representative data from the NSDUH, this study highlighted the patterns and disparities of mental health service utilization among adolescents who experienced MDE in 2022. We found that among adolescent populations with MDE, less than half received any type of mental health treatment during the pandemic. Moreover, there were notable disparities in treatment by rurality, race/ethnicity, gender, and health insurance that may have profound medical and public health implications.

Our findings build upon previous work establishing the long-standing rural/urban gap in mental health service utilization by illustrating this disparity persisted amidst the COVID-19 pandemic [29,32,33]. Unsurprisingly, adolescents in rural areas utilized fewer specialist clinician treatment and telehealth services than in large metropolitan areas, emphasizing the need for rural-specific service providers and increasing the availability of specialized mental healthcare for rural adolescents [34–37]. It has been emphasized that bridging the digital divide is essential for overcoming disparities in rural mental health services, particularly due to the uneven adoption of telehealth in these areas [34]. Data from the U.S. Census Bureau’s American Community Survey indicate that over 75% of households in urban counties have broadband subscriptions, compared to about 65% in predominantly rural counties [38]. Moreover, rural residents often experience lower broadband speed and pay as much as $25 more per month compared to their urban counterparts [39]. Rural adolescents are also less likely compared to urban/suburban peers to own smartphones and rely on shared computers [40]. Improved broadband coverage could make telehealth a viable solution for enhancing access to care and addressing mental health and healthcare disparities in rural areas [41]. A study conducted in rural Pennsylvania analyzing the emergence of telehealth for rural mental health service delivery during the pandemic revealed positive outcomes such as service continuation, decreased no-show rates, increased parental involvement, and ease of transportation difficulties [34]. However, existing literature points out that many telehealth platforms are not well-suited for clinicians and young people, particularly for individuals with disabilities or those who have difficulty focusing [42]. Looking ahead, emerging and innovative technologies, like smartphone apps, online programs, and potentially virtual reality, could significantly improve the quality and accessibility of mental health services for young people [43]. For those who either dislike telehealth or do not have the resources to effectively engage in telehealth care, other modes of delivery for mental health care must be implemented.

The findings of this study underscore the enduring racial disparities in healthcare that continued throughout the COVID-19 pandemic. Consistent with Flores et al. (2023) [25], Asian, Black, and Hispanic adolescents were less likely to receive outpatient services, prescriptive medication, or specialty services compared to their White counterparts. In addition, an increased rate of depression can be attributed in part to the emotional trauma caused by the COVID-19 pandemic. This impact was felt by all adolescents but especially pronounced in racial and minority groups who faced disproportionate rates of COVID-19-related morbidity and mortality [25]. Indeed, Black and Hispanic youth were up to 4.5 times higher than White youth to have a parent or grandparent die due to COVID-19 [44]. In addition, the murder of George Floyd in 2020 and the rise in anti-Asian hatred during the COVID-19 pandemic have increased anxiety, depression, and mistrust in health service systems among Asian Americans and Black Americans [45,46]. The gap in service utilization may be attributed to residential segregation, unequal distribution of health resources, cultural stigma surrounding mental health, and misbeliefs about psychological and pharmaceutical treatment for mental health [47,48]. Moreover, parental perceptions play a crucial role in whether minority adolescents pursue mental health services. Parents’ attitudes toward mental health, their ability to identify issues in their children, and their beliefs about seeking help can significantly impact their children’s access to care, either as barriers or facilitators [49]. This finding highlights the need for a deeper understanding of the considerations related to adolescent mental health treatment among diverse cultural groups. By doing so, culturally competent strategies can be developed to effectively engage racial and ethnic minority youth and their parents in treatment.

We found that female adolescents had higher odds of utilizing mental health services, which aligns with previous literature highlighting the disproportionately lower utilization of mental health services among males compared to female adolescents [50]. This trend is influenced by several factors, including societal norms that frame emotional expression and vulnerability as a weakness and contradict the traditional notion of masculinity that adolescent males often seek to uphold [51]. This stigma can prevent them from openly discussing their feelings, acknowledging their mental health struggles, or seeking help [51]. Consistent with previous studies, this finding stresses the importance of mental health literacy to dispel misconceptions and destigmatize mental health service-seeking among male adolescents [52].

A surprising finding is that compared to adolescents with health insurance, adolescents without any type of insurance coverage had a significantly lower utilization of telehealth services and prescription medication, but not the other types of mental health treatment. The correlation between health insurance gaps and prescription-related issues is well-documented [53]. However, even though previous findings suggest that having health insurance coverage for mental or behavioral health needs had lower odds of experiencing perceived difficulties obtaining care [32], we speculate that communities most critically affected by the loss of employment and health insurance coverage may be turning to safety-net settings like community health centers and federally qualified health centers for mental health treatment. Indeed, federally funded health centers have reported increased pediatric mental health patients during the pandemic but continue to face barriers to telehealth use due to cost, reimbursements, and technical issues [54,55].

Schools can potentially improve accessibility to mental health services, as adolescents spend most of their day in the academic setting. To that end, we found no significant differences in school-based mental health service utilization based on gender, rural-urban areas, and health insurance status. Moreover, only Black adolescents had significantly lower odds of receiving school-based services as compared to non-Hispanic White adolescents, as there was no statistical difference in odds of school-based mental health service utilization between Asian, Hispanic, non-Hispanic White, and adolescents of “Other” racial groups. During the 2021 and 2022 school years, 96% of public schools reported providing mental health services for their students; nevertheless, the most common limitations identified were a shortage of mental health professionals to handle the schools’ caseloads, limited access to licensed mental health professionals, and inadequate funding [56]. Intervention programs with a school-based approach have correlated with a greater utilization rate and lower stigma, specifically within minority groups [57]. These school-based approaches must have the requisite resources to meet the needs of their student body such that they can meet the needs of students from diverse backgrounds. If successful, school-based clinics could bridge the accessibility gap, providing primary and preventative health services to young people living in underserved communities [58]. However, this intervention may not fully address Black youth’s needs, further emphasizing the crucial role of culturally competent approaches to enhance their engagement with school-based services.

Our findings should be considered amid several limitations. First, the NSDUH is a cross-sectional design, which prevents causal inferences. Second, the data relies on adolescents’ self-reporting, making it susceptible to recall and social-desirability biases. Third, the NSDUH data reported major depressive episodes (MDE) within the past year without distinguishing between current and past MDE status or the severity of depression. This limitation hinders the ability to differentiate between ongoing and resolved cases of depression, as well as to assess the severity of depression among adolescents. Fourth, the dataset dichotomized gender as male and female, omitting analysis of mental health service utilization among non-binary and transgender adolescents, who are at a higher risk for mental health issues and encounter greater barriers to treatment [59]. Fifth, the exclusion criteria for the NSDUH sample further limit the generalizability of the findings, as it did not include the individuals who are homeless, incarcerated, or in residential treatment. Consequently, our estimates may be optimistic as these groups are more likely to experience higher levels of depression and face more challenges in accessing mental health treatment. Sixth, due to sample size, we grouped Native American and Alaska Native adolescents as a single race/ethnicity category to ensure sufficient data points for analysis. Lastly, the 2022 NSDUH survey used web-based and in-person self-interview methods to enhance response accuracy, restricting comparisons of mental health utilization patterns and disparities to pre-pandemic years.

## Conclusion

The findings of this national cross-sectional study build upon existing foundational literature [29,32,33], illustrating the persistence of disparities in mental health service utilization, particularly among adolescents from racial and ethnic minority groups, during the third year of the COVID-19 pandemic. Moving forward, policy and clinical initiatives should be informed by further research into how cultural and systemic factors influence the utilization of mental health services and help-seeking behaviors in race/ethnic minority populations. Additionally, gaps in telehealth service utilization indicate that adolescents without health insurance and those from rural areas require increased support to access mental health services available through telecommunications technology effectively. This study also suggested leveraging and expanding school-based mental health services to address access barriers related to gender, race/ethnicity, rurality, and health insurance coverage. In sum, addressing the disparities in mental health treatment revealed by this study is crucial for ensuring a more inclusive and equitable mental health support system for all adolescents.

## Supporting information

S1 Text Primary types of public insurance in the United States. (DOCX)

## References

[1] Wilson S, Dumornay NM. Rising rates of adolescent depression in the United States: challenges and opportunities in the 2020s. J Adolesc Health. 2022;70(3):354–5. doi: 10.1016/j.jadohealth.2021.12.003 35183317 PMC8868033

[2] Substance Abuse and Mental Health Services Administration. National survey on drug use and health. Rockville (MD): SAMHSA; 2022. https://www.samhsa.gov/data/data-we-collect/nsduh-national-survey-drug-use-and-health

[3] Knopf A. NSDUH finds substance use, depression, suicidality high among youth. Child Adolesc Behav. 2023;40(1):5–6. doi: 10.1002/cbl.30759

[4] Chavira DA, Ponting C, Ramos G. The impact of COVID-19 on child and adolescent mental health and treatment considerations. Behav Res Ther. 2022;157:104169. doi: 10.1016/j.brat.2022.104169 35970084 PMC9339162

[5] de Girolamo G, Dagani J, Purcell R, Cocchi A, McGorry PD. Age of onset of mental disorders and use of mental health services: needs, opportunities and obstacles. Epidemiol Psychiatr Sci. 2012;21(1):47–57. doi: 10.1017/s2045796011000746 22670412

[6] Meherali S, Punjani N, Louie-Poon S, Abdul Rahim K, Das JK, Salam RA, et al. Mental health of children and adolescents amidst COVID-19 and past pandemics: a rapid systematic review. Int J Environ Res Public Health. 2021;18(7):3432. doi: 10.3390/ijerph18073432 33810225 PMC8038056

[7] Meade J. Mental health effects of the COVID-19 pandemic on children and adolescents: a review of the current research. Pediatr Clin North Am. 2021;68(5):945–59. doi: 10.1016/j.pcl.2021.05.003 34538305 PMC8445752

[8] Schleider JL, Weisz RJ. Can less be more? The promise (and perils) of single-session youth mental health interventions. Behav Therapist. 2017;40(2):256–61. https://www.researchgate.net/publication/318858941_Can_less_be_more_The_promise_and_perils_of_single-session_youth_mental_health_interventions

[9] McGorry PD, Mei C. Early intervention in youth mental health: progress and future directions. Evid Based Ment Health. 2018;21(4):182–4. doi: 10.1136/ebmental-2018-300060 30352884 PMC10270418

[10] Lin C, Pham H, Hser Y-I. Mental health service utilization and disparities in the U.S: observation of the first year into the COVID pandemic. Community Ment Health J. 2023;59(5):972–85. doi: 10.1007/s10597-022-01081-y 36609783 PMC11329229

[11] Burrell TD, Kim S, Mohadikar K, Jonas C, Ortiz N, Horberg MA. Family structure and adolescent mental health service utilization during the COVID-19 pandemic. J Adolesc Health. 2023;73(4):693–700. doi: 10.1016/j.jadohealth.2023.01.018 37032208 PMC10081921

[12] Mojtabai R, Olfson M, Han B. National trends in the prevalence and treatment of depression in adolescents and young adults. Pediatrics. 2016;138(6):e20161878. doi: 10.1542/peds.2016-1878 27940701 PMC5127071

[13] Berryhill B, Carlson C, Hopson L, Culmer N, Williams N. Adolescent depression and anxiety treatment in rural schools: a systematic review. Rural Ment Health. 2022;46(1):13–27. doi: 10.1037/rmh0000183 37333612 PMC10275338

[14] Alcendor DJ. Racial disparities-associated COVID-19 mortality among minority populations in the US. J Clin Med. 2020;9(8):2442. doi: 10.3390/jcm9082442 32751633 PMC7466083

[15] Mude W, Oguoma VM, Nyanhanda T, Mwanri L, Njue C. Racial disparities in COVID-19 pandemic cases, hospitalisations, and deaths: a systematic review and meta-analysis. J Glob Health. 2021;11:05015. doi: 10.7189/jogh.11.05015 34221360 PMC8248751

[16] Luck AN, Preston SH, Elo IT, Stokes AC. The unequal burden of the Covid-19 pandemic: Capturing racial/ethnic disparities in US cause-specific mortality. SSM Popul Health. 2022;17:101012. doi: 10.1016/j.ssmph.2021.101012 34961843 PMC8697426

[17] Millett GA, Jones AT, Benkeser D, Baral S, Mercer L, Beyrer C, et al. Assessing differential impacts of COVID-19 on black communities. Ann Epidemiol. 2020;47:37–44. doi: 10.1016/j.annepidem.2020.05.003 32419766 PMC7224670

[18] Dos Santos ERR, Silva de Paula JL, Tardieux FM, Costa-E-Silva VN, Lal A, Leite AFB. Association between COVID-19 and anxiety during social isolation: a systematic review. World J Clin Cases. 2021;9(25):7433–44. doi: 10.12998/wjcc.v9.i25.7433 34616809 PMC8464456

[19] Iwata M, Ota KT, Duman RS. The inflammasome: pathways linking psychological stress, depression, and systemic illnesses. Brain Behav Immun. 2013;31:105–14. doi: 10.1016/j.bbi.2012.12.008 23261775 PMC4426992

[20] Woody ML, Bell EC, Cruz NA, Wears A, Anderson RE, Price RB. Racial stress and trauma and the development of adolescent depression: a review of the role of vigilance evoked by racism-related threat. Chronic Stress (Thousand Oaks). 2022;6:24705470221118574. doi: 10.1177/24705470221118574 35966451 PMC9373112

[21] Reck AJ, Kogan SM. Family stress and rural african-american adolescents’ depressive symptoms. J Adolesc Health. 2021;69(6):1006–12. doi: 10.1016/j.jadohealth.2021.05.005 34092476 PMC8612945

[22] Andrilla CHA, Patterson DG, Garberson LA, Coulthard C, Larson EH. Geographic variation in the supply of selected behavioral health providers. Am J Prev Med. 2018;54(6 Suppl 3):S199–207. doi: 10.1016/j.amepre.2018.01.004 29779543

[23] Summers-Gabr NM. Rural-urban mental health disparities in the United States during COVID-19. Psychol Trauma. 2020;12(S1):S222–4. doi: 10.1037/tra0000871 32478541

[24] Kataoka SH, Zhang L, Wells KB. Unmet need for mental health care among U.S. children: variation by ethnicity and insurance status. Am J Psychiatry. 2002;159(9):1548–55. doi: 10.1176/appi.ajp.159.9.1548 12202276

[25] Flores MW, Sharp A, Carson NJ, Cook BL. Estimates of major depressive disorder and treatment among adolescents by race and ethnicity. JAMA Pediatr. 2023;177(11):1215–23. doi: 10.1001/jamapediatrics.2023.3996 37812424 PMC10562990

[26] Banks A. Black adolescent experiences with COVID-19 and mental health services utilization. J Racial Ethn Health Disparities. 2022;9(4):1097–105. doi: 10.1007/s40615-021-01049-w 33909283 PMC8080856

[27] Burrell TD, Sheu Y-S, Kim S, Mohadikar K, Ortiz N, Jonas C, et al. COVID-19 and adolescent outpatient mental health service utilization. Acad Pediatr. 2024;24(1):68–77. doi: 10.1016/j.acap.2023.05.016 37302698 PMC10250250

[28] Ali MM, Sherman LJ, Lynch S, Teich J, Mutter R. Differences in utilization of mental health treatment among children and adolescents with medicaid or private insurance. Psychiatr Serv. 2019;70(4):329–32. doi: 10.1176/appi.ps.201800428 30691383

[29] Lu W. Adolescent depression: national trends, risk factors, and healthcare disparities. Am J Health Behav. 2019;43(1):181–94. doi: 10.5993/AJHB.43.1.15 30522576

[30] Merikangas KR, Avenevoli S, Costello EJ, Koretz D, Kessler RC. National comorbidity survey replication adolescent supplement (NCS-A): I. Background and measures. J Am Acad Child Adolesc Psychiatry. 2009;48(4):367–79. doi: 10.1097/CHI.0b013e31819996f1 19242382 PMC2736858

[31] United States Department of Agriculture Economic Research Service. Rural-urban continuum codes. Washington (DC): USDA ERS; 2023. https://www.ers.usda.gov/data-products/rural-urban-continuum-codes.aspx

[32] Mahmood A, Kedia S, Arshad H, Mou X, Dillon PJ. Disparities in access to mental health services among children diagnosed with anxiety and depression in the United States. Community Ment Health J. 2024;60(8):1532–46. doi: 10.1007/s10597-024-01305-3 38907843 PMC11579094

[33] Hoffmann JA, Alegría M, Alvarez K, Anosike A, Shah PP, Simon KM, et al. Disparities in pediatric mental and behavioral health conditions. Pediatrics. 2022;150(4):e2022058227. doi: 10.1542/peds.2022-058227 36106466 PMC9800023

[34] Nelson D, Inghels M, Kenny A, Skinner S, McCranor T, Wyatt S, et al. Mental health professionals and telehealth in a rural setting: a cross sectional survey. BMC Health Serv Res. 2023;23(1):200. doi: 10.1186/s12913-023-09083-6 36849933 PMC9970689

[35] Anderson RL, Gittler J. Unmet need for community-based mental health and substance use treatment among rural adolescents. Community Ment Health J. 2005;41(1):35–49. doi: 10.1007/s10597-005-2598-0 15932051

[36] Morales DA, Barksdale CL, Beckel-Mitchener AC. A call to action to address rural mental health disparities. J Clin Transl Sci. 2020;4(5):463–7. doi: 10.1017/cts.2020.42 33244437 PMC7681156

[37] Anderson JK, Howarth E, Vainre M, Jones PB, Humphrey A. A scoping literature review of service-level barriers for access and engagement with mental health services for children and young people. Chil Youth Serv Rev. 2017;77:164–76. doi: 10.1016/j.childyouth.2017.04.017

[38] U.S. Census Bureau. For the first time, Census Bureau data show impact of geography, income on broadband internet access. U.S. Census Bureau. 2018 [Accessed 2025 January 10]. https://www.census.gov/library/stories/2018/12/rural-and-lower-income-counties-lag-nation-internet-subscription.html

[39] Whitacre B. Research and analysis: rural internet subscribers pay more, new data confirms. Daily Yonder. 2023. https://dailyyonder.com/research-and-analysis-rural-internet-subscribers-pay-more-new-data-confirms/2023/11/28/

[40] Madden M. Main findings. 2013 [Accessed 2025 January 10]. https://www.pewresearch.org/internet/2013/03/13/main-findings-5

[41] Graves JM, Abshire DA, Amiri S, Mackelprang JL. Disparities in technology and broadband internet access across rurality: implications for health and education. Fam Community Health. 2021;44(4):257–65. doi: 10.1097/FCH.0000000000000306 34269696 PMC8373718

[42] Nicholas J, Bell IH, Thompson A, Valentine L, Simsir P, Sheppard H, et al. Implementation lessons from the transition to telehealth during COVID-19: a survey of clinicians and young people from youth mental health services. Psychiatry Res. 2021;299:113848. doi: 10.1016/j.psychres.2021.113848 33725578 PMC9754759

[43] Bell IH, Nicholas J, Alvarez-Jimenez M, Thompson A, Valmaggia L. Virtual reality as a clinical tool in mental health research and practice. Dialogues Clin Neurosci. 2020;22(2):169–77. doi: 10.31887/DCNS.2020.22.2/lvalmaggia 32699517 PMC7366939

[44] Hillis SD, Blenkinsop A, Villaveces A, Annor FB, Liburd L, Massetti GM, et al. COVID-19-associated orphanhood and caregiver death in the United States. Pediatrics. 2021;148(6):e2021053760. doi: 10.1542/peds.2021-053760 PMC10896160 34620728

[45] Alang SM. Mental health care among blacks in America: confronting racism and constructing solutions. Health Serv Res. 2019;54(2):346–55. doi: 10.1111/1475-6773.13115 30687928 PMC6407345

[46] Eichstaedt JC, Sherman GT, Giorgi S, Roberts SO, Reynolds ME, Ungar LH, et al. The emotional and mental health impact of the murder of George Floyd on the US population. Proc Natl Acad Sci U S A. 2021;118(39):e2109139118. doi: 10.1073/pnas.2109139118 34544875 PMC8488615

[47] Ahad AA, Sanchez-Gonzalez M, Junquera P. Understanding and addressing mental health stigma across cultures for improving psychiatric care: a narrative review. Cureus. 2023;15(5):e39549. doi: 10.7759/cureus.39549 37250612 PMC10220277

[48] Williams DR, Collins C. Racial residential segregation: a fundamental cause of racial disparities in health. Public Health Rep. 2001;116(5):404–16. doi: 10.1093/phr/116.5.404 12042604 PMC1497358

[49] Lu W, Todhunter-Reid A, Mitsdarffer ML, Muñoz-Laboy M, Yoon AS, Xu L. Barriers and facilitators for mental health service use among racial/ethnic minority adolescents: a systematic review of literature. Front Public Health. 2021;9:641605. doi: 10.3389/fpubh.2021.641605 33763401 PMC7982679

[50] Liddle SK, Vella SA, Deane FP. Attitudes about mental illness and help seeking among adolescent males. Psychiatry Res. 2021;301:113965. doi: 10.1016/j.psychres.2021.113965 34023672

[51] Randell E, Jerdén L, Öhman A, Starrin B, Flacking R. Tough, sensitive and sincere: how adolescent boys manage masculinities and emotions. Int J Adolesc Youth. 2015;21(4):486–98. doi: 10.1080/02673843.2015.1106414

[52] Freţian AM, Graf P, Kirchhoff S, Glinphratum G, Bollweg TM, Sauzet O, et al. The long-term effectiveness of interventions addressing mental health literacy and stigma of mental illness in children and adolescents: systematic review and meta-analysis. Int J Public Health. 2021;66:1604072. doi: 10.3389/ijph.2021.1604072 34975363 PMC8714636

[53] Seo V, Baggett TP, Thorndike AN, Hull P, Hsu J, Newhouse JP, et al. Access to care among Medicaid and uninsured patients in community health centers after the Affordable Care Act. BMC Health Serv Res. 2019;19(1):291. doi: 10.1186/s12913-019-4124-z 31068205 PMC6505197

[54] Lee CM, Lutz J, Khau A, Lin B, Phillip N, Ackerman S, et al. Pediatric primary care perspectives of mental health services delivery during the COVID-19 pandemic. Children (Basel). 2022;9(8):1167. doi: 10.3390/children9081167 36010056 PMC9406881

[55] Lin C-CC, Dievler A, Robbins C, Sripipatana A, Quinn M, Nair S. Telehealth in health centers: key adoption factors, barriers, and opportunities. Health Aff (Millwood). 2018;37(12):1967–74. doi: 10.1377/hlthaff.2018.05125 30633683

[56] Institute of education sciences. Washington, DC: U.S. Department of Education; 2022. https://ies.ed.gov/schoolsurvey/spp/

[57] Stephan SH, Weist M, Kataoka S, Adelsheim S, Mills C. Transformation of children’s mental health services: the role of school mental health. Psychiatr Serv. 2007;58(10):1330–8. doi: 10.1176/ps.2007.58.10.1330 17914011

[58] Mason-Jones AJ, Crisp C, Momberg M, Koech J, De Koker P, Mathews C. A systematic review of the role of school-based healthcare in adolescent sexual, reproductive, and mental health. Syst Rev. 2012;1:49. doi: 10.1186/2046-4053-1-49 23098138 PMC3621403

[59] Lothwell LE, Libby N, Adelson SL. Mental health care for LGBT youths. Focus (Am Psychiatr Publ). 2020;18(3):268–76. doi: 10.1176/appi.focus.20200018 33162863 PMC7587912

