# Supplementary material for: Disparities in mental health service utilization among adolescents with depression: Results from a 2022 U.S. National Survey
Source: PLOS Ment Health. 2025 Aug 20;2(8):e0000388. doi: 10.1371/journal.pmen.0000388 (PMC12798591; doi:10.1371/journal.pmen.0000388)
Supplement: S1 Text — (DOCX) [file pmen.0000388.s001.docx]

**Supplemental Material: Primary Types of Public Insurance in the United States**

There are three primary types of public insurance in the United States for which children (aged 18 years or less) are eligible. Medicaid is available to children whose families fall below the federal poverty line, and the services covered by Medicaid differ across states as states are the primary administrators of Medicaid. However, many families may have earnings above the federal poverty line but do not have access to health insurance through their employer or may not be able to afford private health insurance. Children from those families may receive health insurance through the Children’s Health Insurance Program. Eligibility and benefits vary across states because, like Medicaid, states are the primary administrators of the Children’s Health Insurance Program. Family income must typically fall between 200-300% of the federal poverty line for children to be eligible for this program. Lastly, Medicare is a federally administered health insurance program that predominately provides insurance to individuals aged 65 years or more, those with end-stage renal disease, or those with a disability. Children may receive insurance through Medicare if they have end-stage renal disease or if they are between the ages of 20-22 years and their guardian receives Social Security benefits or has earned a requisite number of Social Security credits. In addition to public programs, families in the U.S. may secure health coverage for their children through private insurance, either via employer-sponsored plans or by purchasing policies independently.

Reference: Kalainov DM, Yamaguchi K. Medicaid and the Children's Health Insurance Program: Understanding These Programs to Promote Advancements. J Am Acad Orthop Surg. 2025 Feb 1;33(3):117-126. doi: 10.5435/JAAOS-D-23-00735. Epub 2024 Oct 8. PMID: 39822079; PMCID: PMC11708994.
